# Isolation and characterization of a Sca-1^+^/CD31^-^ progenitor cell lineage derived from mouse heart tissue

**DOI:** 10.1186/1472-6750-14-75

**Published:** 2014-08-09

**Authors:** Hao Wang, Hao Chen, Bei Feng, Xiang Wang, Xiaomin He, Renjie Hu, Meng Yin, Wei Wang, Wei Fu, Zhiwei Xu

**Affiliations:** 1 Department of Pediatric Cardiothoracic Surgery, Shanghai Children's Medical Center, School of Medicine, Shanghai Jiao Tong University, 1678 Dong Fang Road, Shanghai 200127, China; 2 Institute of Pediatric Translational Medicine, Shanghai Children's Medical Center, School of Medicine, Shanghai Jiao Tong University, 1678 Dong Fang Road, Shanghai 200127, China; 3 Department of Hematology/Oncology, Shanghai Children's Medical Center, School of Medicine, Shanghai Jiao Tong University, 1678 Dong Fang Road, Shanghai 200127, China

**Keywords:** Cardiac progenitor cell, Stem cell antigen-1, Differentiation, Multipotent, Self-renewal

## Abstract

**Background:**

Myocardial infarction remains the leading cause of mortality in developed countries despite recent advances in its prevention and treatment. Regenerative therapies based on resident cardiac progenitor cells (CPCs) are a promising alternative to conventional treatments. However, CPCs resident in the heart are quite rare. It is unclear how these CPCs can be isolated and cultured efficiently and what the effects of long-term culture in vitro are on their ‘stemness’ and differentiation potential, but this is critical knowledge for CPCs’ clinical application.

**Results:**

Here, we isolated stem cell antigen-1 positive cells from postnatal mouse heart by magnetic active cell sorting using an iron-labeled anti-mouse Sca-1 antibody, and cultured them long-term in vitro. We tested stemness marker expression and the proliferation ability of long-term cultured Sca-1^+^ cells at early, middle and late passages. Furthermore, we determined the differentiation potential of these three passages into cardiac cell lineages (cardiomyocytes, smooth muscle and endothelial cells) after induction in vitro. The expression of myocardial, smooth muscle and endothelial cell-specific genes and surface markers were analyzed by RT-PCR and IF staining. We also investigated the oncogenicity of the three passages by subcutaneously injecting cells in nude mice. Overall, heart-derived Sca-1^+^ cells showed CPC characteristics: long-term propagation ability in vitro, non-tumorigenic in vivo, persistent expression of stemness and cardiac-specific markers, and multipotent differentiation into cardiac cell lineages.

**Conclusions:**

Our research may bring new insights to myocardium regeneration, for which even a small number of biopsy-derived CPCs could be enriched and propagated long term in vitro to obtain sufficient seed cells for cell injection or cardiac tissue engineering.

## Background

Despite recent progress in prevention and therapies, heart failure resulting from acute myocardial infarction or chronic myocardial ischemia remains the leading cause of mortality in developed countries [1]. The central cellular mechanism underlying myocardial dysfunction is an irreversible loss of viable cardiomyocytes and an inability of residual cardiomyocytes to compensate for this loss [2,3]. Heart transplantation remains the final therapeutic option for end-stage heart failure [4], but is limited by donor organ shortages and life-threatening complications, including organ rejection and side effects of pharmacological immune suppression. Regenerative therapies, including cell injection and myocardial tissue engineering, have been pursued as new possibilities to repair the damaged myocardium. Over the last decade, much research has focused on finding the ideal cell type to mediate myocardial repair. To date, cells derived from fetal or neonatal hearts have been applied to damaged myocardia to regenerate myocardial tissues in animal studies [5,6], but a similar approach in humans poses ethical issues and may be insufficient to provide the large number of cardiomyocytes needed for clinical use [7]. Developing methods to use stem cell-derived cardiomyocytes may be required.

The recent discovery of resident cardiac progenitor cells (CPCs) in the postnatal heart has marked a new era of cardiac regenerative medicine. Several laboratories have identified distinct populations of CPCs including side population (SP) cells [8], stem cell antigen-1-positive (Sca-1^+^) cells [9], c-kit-positive (c-kit^+^) cells [10], Islet-1-positive (Isl-1^+^) cells [11,12], Wilms’ tumor 1-positive (WT1^+^) epicardial progenitor cells [13], and cardiosphere-derived cells (CDCs) [14]. However, the levels of resident CPCs in postnatal heart are limited and there is a clear negative correlation between CPC number and age [15]. Furthermore, in clinical practice, CPCs may be more likely to be isolated from catheter-based tiny biopsy specimens rather than large masses of cardiac tissue. Yet, large quantities of CPCs are needed for myocardium regeneration.

Goumans’ team has developed a method for efficient isolation and expansion of human CPCs from cardiac surgical waste by magnetic cell sorting (MACS) and has developed a detailed protocol for very efficient in vitro differentiation of CPCs into cardiomyocytes (80–90% differentiation) [16]. However, it is unclear how many seed cells would be eventually obtained for regenerative therapies and whether their ‘stemness’ or differentiation potential would decline with long-term culture in vitro. In this report, we isolated Sca-1^+^ cells from postnatal mouse heart by MACS using an iron-labeled anti-mouse Sca-1 antibody, and cultured the cells long-term in vitro. To monitor the stemness and differentiation potential of long-term cultured Sca-1^+^ cells, we selected the 7th, 28th, and 53th passages to test early, middle and late phases of culture. We further identified these cells as Sca-1^+^/CD31^-^ CPC subpopulations, which are able to proliferate over the long-term in vitro without a detectable decline in stemness. In addition, they retain the potential to differentiate into multiple cardiac cell lineages.

## Results

### Isolation and in vitro culture of postnatal mouse heart-derived Sca-1^+^ cells

**Figure 1:**
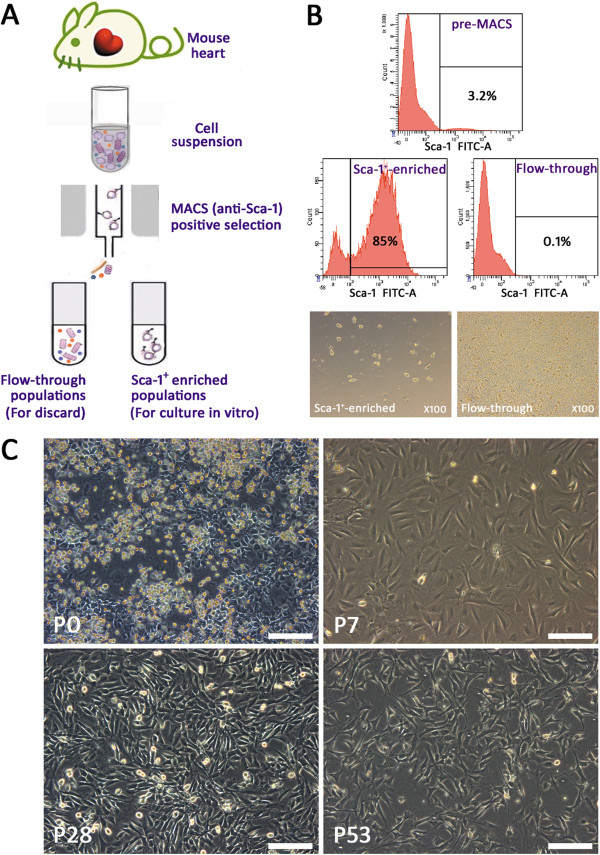
**Isolation and enrichment of Sca-1**^**+ **^**cells from the mouse heart and in vitro culture. A**. Schematic representation of the isolation and enrichment of Sca-1^+^ cell populations from the mouse heart. **B**. Efficiency and feasibility of MACS. The proportion of Sca-1^+^ cells contained in pre-MACS population, Sca-1^+^-enriched population, and flow-through population analyzed by flow cytometry. The lower two panels show the morphology of cells after MACS under ordinary light microscopy (×100). **C**. Cells of Sca-1^+^-enriched populations cultured and passaged in vitro. Morphology of cells under inverted microscopy. Scale bar = 200 μm.

MACS is an efficient system for cell isolation, especially for rare cells in tissues [17]. We used this system to isolate resident stem cells from postnatal mice hearts, based on the expression of the surface marker stem cell antigen Sca-1 (Figure 1A). To verify the efficiency of MACS using an iron-labeled anti-Sca-1 antibody, we performed FCM to analyze the enrichment of Sca-1^+^ cells in samples before and after MACS. Pre-MACS, just after filtration through a 40-μm cell strainer, the whole cardiac cell suspension was composed of approximately 3.2% Sca-1^+^ cells. After MACS, the Sca-1^+^-enriched population contained approximately 85% Sca-1^+^ cells, and the flow-through population contained only 0.1% Sca-1^+^ cells (Figure 1B). Viewed with an inverted microscopy in ordinary light, cells in Sca-1^+^-enriched populations were sparse, with quasi-circular or oval morphology and homogenous in size, while cells in flow-through populations were crowded, containing diverse cell types and sizes (Figure 1B). Thus, the efficiency and specificity of MACS were considered reasonable.

After seeding on gelatin-coated 12-well dishes, the long-term cell culture of Sca-1^+^-enriched population was initiated. After about one week, small, flat, spindle-shaped cells appeared and grew gradually. Many of these cells were phase-bright under white light. On day 36, the primary cells (P0) had grown to approximately 90% confluence and were propagated to P1 at 1:2, that is, transferred from one well of the 12-well dish (4.5 cm^2^) to one well in a 6-well dish (9.6 cm^2^), also coated with gelatin. About 4 days after the first passage, cells of P1 were detached and further subcultured as P2 at 1:5; cells were transferred from one well of the 6-well dish (9.6 cm^2^) to one 9-cm dish (49 cm^2^) without gelatin coating. P2 cells were cultured in normal medium for 4 weeks, at which point they reached 90% confluence and were split at 1:2 to P3. P3 grew more rapidly and was split after about 1 week. From then on, the subcultured cells were passaged every 3–4 days at different ratios. The Sca-1^+^ cells were passaged to over P90 and still grew well with phase-brightness (data not shown). There were no noticeable changes in gross morphology until at least P53 (representing a period of 245 days; Figure 1C).

### Surface characteristics of Sca-1^+^-enriched long-term cell culture

**Figure 2:**
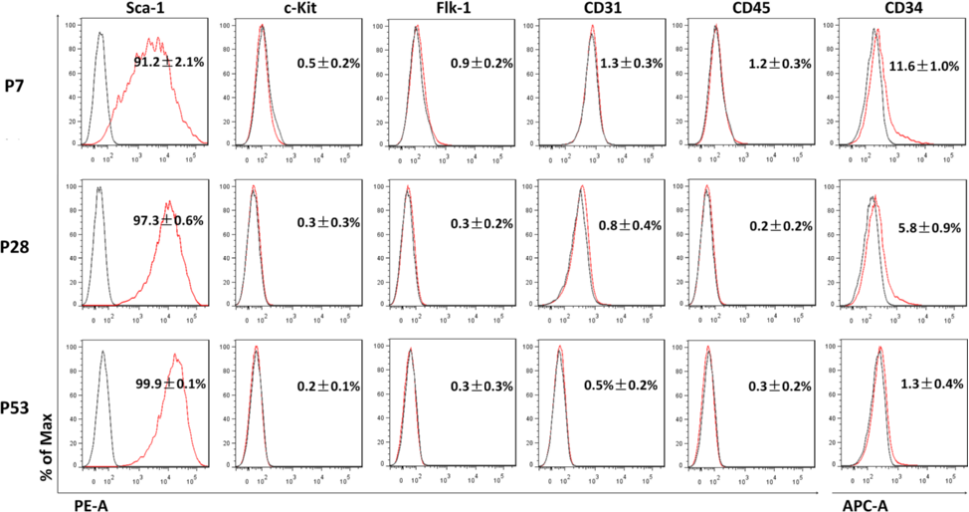
**Surface marker expression profiles of three subcultures derived from Sca-1**^**+**^**-enriched populations analyzed by flow cytometry.** All populations were analyzed for the expression of the stem/progenitor cell markers Sca-1 and c-kit, endothelial progenitor cell marker flk-1, endothelial cell specific marker CD31, hematopoietic progenitor marker CD34, and hematopoietic lineage marker CD45. Black line, non-stained cells; red line, corresponding antibody. (n = 3).

To identify the surface characteristics of heart-derived Sca-1^+^-enriched cells and determine whether these would change during long-term in vitro culture, cells of P7, P28, and P53 were analyzed for stem cell antigen Sca-1 and other cell surface markers by FCM and IF staining. FCM analysis showed that the percentage of Sca-1^+^ cells increased from 91.2% to 99.9% over P7 to P53. This indicates that not only do the Sca-1^+^-enriched passages still mainly consist of Sca-1^+^ cells, but the purity of Sca-1^+^ cells increases with passaging. Few cells were detected that expressing c-kit, another stem cell marker; proportions varied from 0.3-0.6%. Flk-1, an endothelial progenitor cell marker, was also barely expressed. CD34, a hematopoietic progenitor cell marker [18], was initially expressed in 11.6% of P7 cells, which declined to 1.3% of cells at P53. CD31, a marker of endothelial cells [19,20] and CD45, a marker of the hematopoietic lineage [17] were also both rarely expressed (Figure 2).

**Figure 3:**
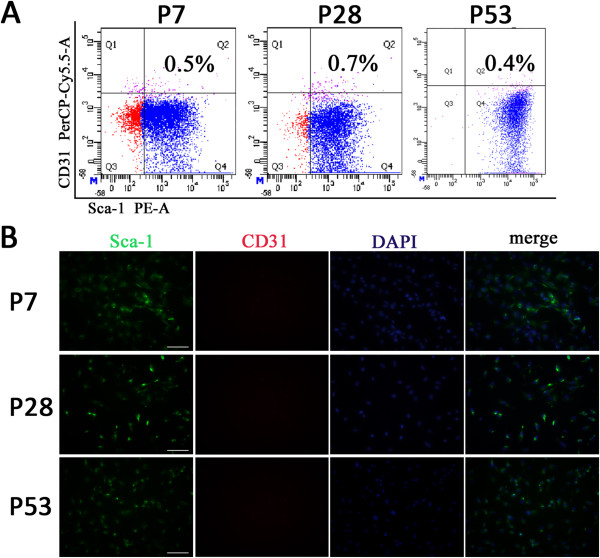
**Analysis of CD31 expression in Sca-1**^**+ **^**cells by FCM and identification of Sca-1**^**+**^**-enriched cells by IF staining. A**. Flow cytometry analyzed the expression of surface markers CD31 in Sca-1^+^ cells. **B**. All cells were triple-stained for Sca-1 (green), CD31 (red), and DAPI (blue). Bar = 100 μm.

To further characterize the Sca-1^+^ cells, we double labeled them with antibodies against Sca-1 and CD31 (Figure 3), because Sca-1^+^/CD31^-^ CPCs show functional cardiomyogenic differentiation, and Sca-1^+^/CD31^+^ cells do not [21]. Based on FCM, there was strong expression of Sca-1 in cells at P7, P28, and P53, but few was Sca-1^+^/CD31^+^ cells (Figure 3A), which was confirmed by IF staining. IF staining showed that few cells expressed CD31 in Sca-1^+^ enriched cell populations (Figure 3B). These data indicate that the mouse heart-derived Sca-1^+^ cells isolated here represent a Sca-1^+^/CD31^-^ subpopulation.

### Gene expression profile of Sca-1^+^-enriched cells

**Figure 4:**
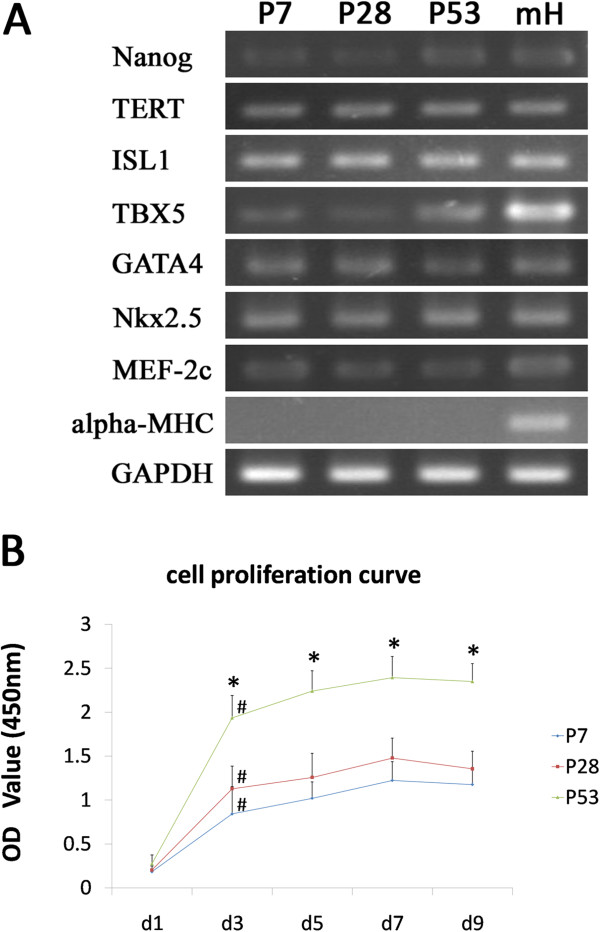
**Gene expression profiles and proliferation abilities of Sca-1 high-expressing cell populations analyzed by RT-PCR and CCK-8. A**. Expression of stem cell-related genes Nanog and TERT, cardiac precursor-related genes ISL-1 and TBX5, early cardiogenic genes GATA4, Nkx2.5, MEF2c, and mature cardiomyocyte specific gene α-MHC was analyzed by RT-PCR. RNA extracted from whole postnatal mouse heart (mH) was used as a positive control. (n = 3). **B**. Proliferation abilities were tested with CCK-8. Five successive time points were chosen: days 1 (24 hours after seeding), 3, 5, 7, and 9. Proliferation abilities of these cell populations were indirectly measured by the OD value at 450 nm. (n = 3). (^*^P < 0.01 vs P7, P28; ^#^*P* < 0.01 vs d1).

To further characterize gene expression in the long-term cultured Sca-1^+^-enriched cells, gene expression profiles of P7, P28, and P53 were generated by RT-PCR. Two pluripotent stem cell specific genes, Nanog and telomerase reverse transcriptase (TERT) [17], were both expressed; TERT is usually not detectable in cardiac fibroblasts [22]. Islet-1 (ISL-1), a marker of the secondary heart field [15], and TBX5, a marker of the primary heart field [17] were both detected, though the expression level of TBX5 was weaker than that of ISL-1. The cardiac-specific transcription factors GATA-4, Nkx-2.5, and MEF2C were all expressed in three different passages. In contrast, the mature cardiomyocyte structural gene cardiac α-myosin heavy chain (α-MHC) [17] was undetectable (Figure 4A). These results indicate that the long-term cultured cardiac-derived Sca-1^+^-enriched cells show cardiac-specific features, but are not mature cardiomyocytes or cardiac fibroblasts.

### In vitro proliferation capacity and tumorigenicity in vivo

The ability to proliferate is a significant feature of stem cells and progenitor cells. The proliferative abilities of P7, P28, and P53 cells were indirectly assessed by measuring the OD value of the supernatant incubated with CCK-8. Five sequential time points were chosen for testing: days 1 (24 hours after seeding), 3, 5, 7 and 9. At day 1 of each passage, the OD value was generally low and there was no statistical difference among these three passages. But from day 1 to day 3, it sharply increased. After day3, the OD value continued increasing slowly, peaked at day 7, then plateaued at this level for several days (Figure 4B). There was no statistical difference in proliferation between P7 and P28. However, the OD value at day 3, day 5, day 7, and day 9 of P53 was significantly higher than that of P7 and P28(*p < 0.01). And in all the comparisons between two adjacent time points in each passage, only day 1 and day 3 presented statistical difference (^#^p < 0.01). These data indicate that the proliferative ability has increased during long term culture, and the significant time point is day 3.

To exclude that Sca-1^+^ cells were spontaneously transforming into tumorigenic cells, cells from P7, P28, and P53 were injected subcutaneously into SCID mice. Equivalent mouse R_1_ ES cells were injected contralaterally as a positive control. No tumors developed at the site of implantation of P7, P28, or P53 cells over approximately 3 months. In contrast, sites injected with R_1_ ES cells all developed neoplasms about 3 weeks after injection; 3 months later, these tumors were nearly 20 mm in diameter. The tumorigenic rates are shown in Additional file 1: Table S1.

### Multipotent differentiation of mouse heart-derived Sca-1^+^ cells into cardiac cell lineages

To estimate the differentiation potential of cells into the cardiomyocyte, smooth muscle, and endothelial lineages, P7, P28, and P53 cells were cultured in specific differentiation medium, with simultaneously cultured in differentiation medium without growth factors serving as controls. When induction was completed, lineage-specific markers were analyzed by IF staining and RT-PCR.

**Figure 5:**
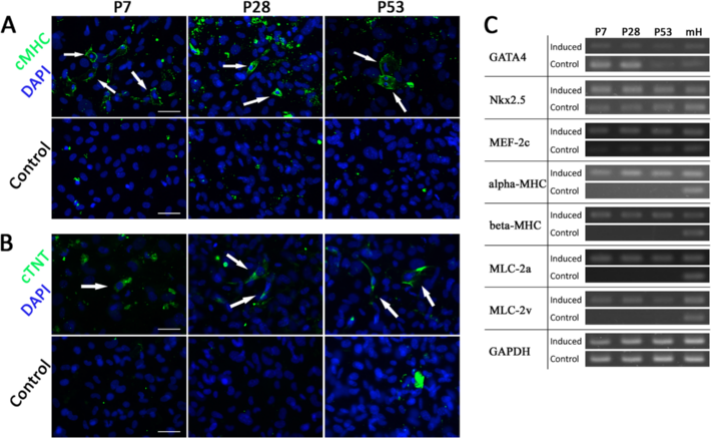
**Differentiation potential of subcultured cells from Sca-1**^**+**^**-enriched populations into cardiomyocyte-like cells in vitro. A-B**. All cell populations were stained for the cardiomyocyte specific marker cardiac MHC (green), cardiac Troponin T (green) and DAPI (blue) after induction. White arrows indicate representative cells positive for each cardiomyocyte-specific antigen. Scale bar = 50 μm. **C**. Expression of cardiac-specific transcription factors GATA4 and Nkx2.5, early cardiogenic gene MEF2c, and cardiomyocyte-specific genes α-MHC, β-MHC, MLC-2a, and MLC-2v was analyzed by RT-PCR. RNA extracted from whole postnatal mouse heart (mH) was used as a positive control.

After 2 weeks of induction to a cardiomyocyte fate, cMHC-positive and cTNT-positive cells were detected in all three passages, and no cMHC- or cTNT-positive cells were observed in control groups (Figure 5A,B). Though the differentiation potential in quantitative analysis of these three passages didn’t present statistical difference among them, it was still low in each passage (Additional file 2: Figure S1A). This cardiomyogenic potential was confirmed by their gene expression profiles. Mature cardiomyocyte marker genes, including cardiac α-myosin heavy chain (α-MHC), cardiac β-myosin heavy chain (β-MHC), myosin light chain-2a (MLC-2a) and myosin light chain-2v (MLC-2v) were expressed in induced cells. In contrast, these genes were not expressed in control groups. After differentiation, the cardiac-specific transcription factor GATA4 was downregulated, while Nkx2.5 and MEF2C were upregulated (Figure 5C).

**Figure 6:**
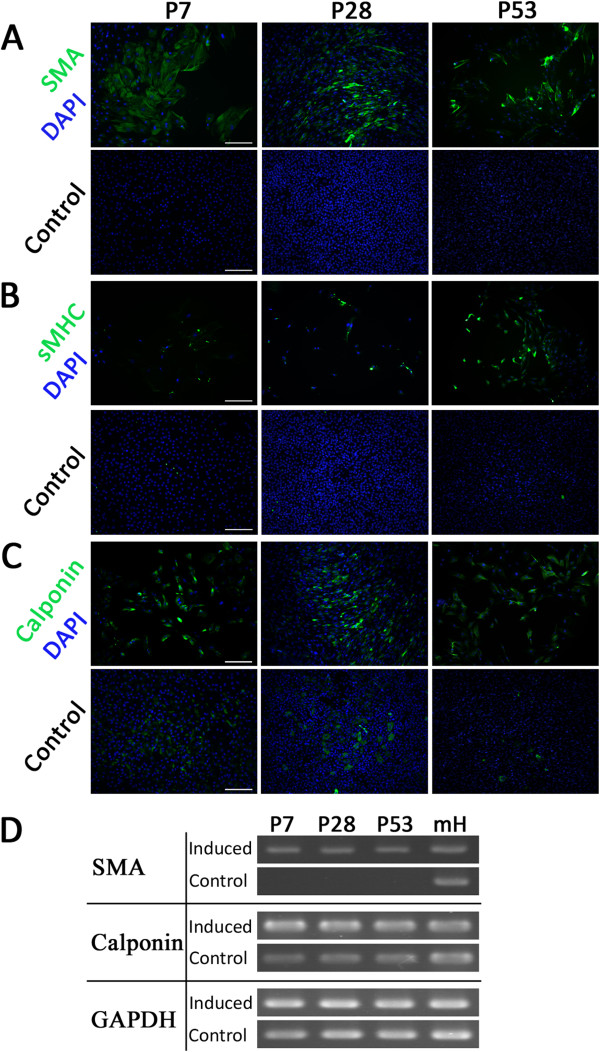
**Differentiation potential of subcultured cells from Sca-1**^**+**^**-enriched population into smooth muscle-like cells in vitro. A-C**. All cell populations were stained for the smooth muscle specific marker SMA (green), smooth muscle MHC (green), calponin (green) and DAPI (blue) after induction. Scale bar = 200 μm. **D**. Expression of smooth muscle specific genes α-SMA and calponin was analyzed by RT-PCR. RNA extracted from whole postnatal mouse heart (mH) was used as a positive control.

After 10 days of induction to a smooth muscle cell fate, SMA-positive and sMHC-positive cells were detected and the number of calponin-positive cells increased significantly compared to the control groups (Figure 6A-C). And the differentiation potential in quantitative analysis of these three passages didn’t present statistical differences among them (Additional file 2: Figure S1B). This result was further verified by examining the gene expression of SMA and calponin. SMA was expressed in all induced passages and there was no expression in control groups, while the expression of calponin was upregulated after induction (Figure 6D).

**Figure 7:**
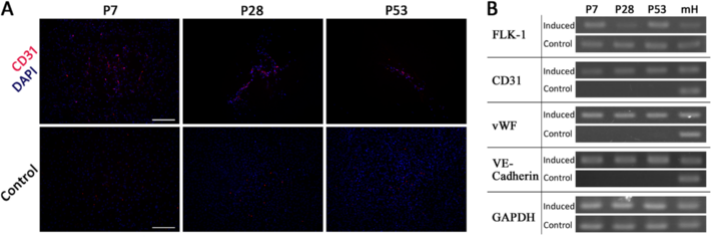
**Differentiation potential of subcultured cells from Sca-1**^**+**^**-enriched populations into endothelial-like cells in vitro. A**. All cell populations were stained for the endothelial cell marker CD31 (red) and DAPI (blue) after induction. Scale bar = 200 μm. **B**. Expression of the endothelial progenitor gene flk-1 and the endothelial cell specific genes CD31, vWF, and VE-Cadherin was analyzed by RT-PCR. RNA extracted from whole postnatal mouse heart (mH) was used as a positive control.

After 2 weeks of induction to an endothelial fate, CD31-positive cells were detected in all passages, while none were detected in control groups (Figure 7A). And the differentiation efficiency of these three passages didn’t present statistical differences among them (Additional file 2: Figure S1C). There was expression of the endothelial specific genes CD31, VE-cadherin, and vWF in all the induced populations, and none in the control groups. The expression level of the endothelial progenitor cell marker Flk-1 did not detectably change after differentiation (Figure 7B). Overall, these data indicate that mouse heart-derived Sca-1^+^ cells are multipotent, retaining an ability to differentiate into different cardiac cell lineages, despite long-term propagation in vitro.

## Discussion

In this article, we successfully isolate Sca-1^+^ cells from postnatal mice hearts with the MACS system, using a microbead-labeled anti-Sca-1 antibody. The isolated Sca-1^+^ cells express the stemness markers Nanog and TERT, the cardiac mesoderm markers ISL-1, TBX5, and the cardiac specific transcription factors GATA4, Nkx2.5, and MEF2C. They can be propagated in vitro for a long time without any significant changes in marker expression. Moreover, the isolated Sca-1^+^ cells and their long-term descendants exhibit multipotent differentiation to cardiac cell lineages, including smooth muscle, endothelial cells and cardiomyocytes. So, the Sca-1^+^ cells we isolated from mouse heart tissue should be identified as CPCs.

Isolation of a cardiac progenitor cell (CPC) population from the heart is challenging, particularly because of a lack of specific surface markers. Though Sca-1^+^ cells, c-kit^+^ cells, Isl-1^+^ cells, WT1^+^ cells, SP cells and CDCs have been extracted from postnatal cardiac tissue [8-14], it remains to be determined whether these individual CPC populations represent different intermediate stages of a single cell lineage or are independent cell lineages originating from different precursors. Some cell markers that could distinguish different lineages, such as Isl-1 and WT-1, are transcription factors, and therefore, transgenic animals would be necessary to genetically tag these proteins to use them as tools to isolate live cells [12,13]. Whether CDCs possess cardiomyogenic potential remains controversial [14,23,24]. Although c-kit^+^ cardiac stem cells were the first stem cells identified in the rat heart [10], recent studies have suggested that Sca-1^+^ progenitor cells are actually the predominant stem cell population there [9,25]: Sca-1^+^ cells are 100–700 fold more frequent than c-kit^+^ cells [10,26]. Although the biological hierarchy and correlation between cardiac SP cells and Sca1^+^ cells is still unclear, interestingly, a high percentage (>80%) of cardiac SP cells express the Sca1 antigen and only a very small percentage (approximately 3.6%) of cardiac Sca-1^+^ cells is included in the SP fraction [9,21]. Furthermore, cardiac SP cells typically lack c-kit, in contrast to marrow SP cells [9,21]. For these reasons, Sca-1^+^ cells in cardiac tissue may be the most populous CPCs or predominate over the long-term, and thus may be relatively easy to isolate from cardiac tissue. This is why we aimed to isolate Sca-1^+^ CPCs from the mouse heart in our research. In 3- to 4-week-old mice, Sca-1^+^ cells accounted for approximately 3.2% of total cardiac cells (Figure 1B), in line with the previously reported 3.0% [27] and 2.1% [9] of Sca-1^+^ cells in 8-week-old and 6- to 12- week-old mice heart respectively; there is a clear negative correlation between CSC number and age [15].

Here, Sca-1^+^ cardiac cells, including early, middle and late passages, showed limited expression of the common stemness marker c-Kit and the hematopoietic lineage markers CD34 and CD45, which characterize bone marrow-derived stem cells [21]. Moreover, they also showed limited expression of the endothelial lineage markers Flk-1 and CD31. However, during early stages of in vitro culture, such as P7, the expression of CD34 in enriched cells is somewhat higher than found in previous studies [17]. We ascribe this outcome to only one round of magnetic sorting. Since CD34 has been characterized as a marker of progenitor cells in several lineages, it may represent another subpopulation of CPC or yet another lineage. Based on FCM and IF staining (Figure 3), these cardiac progenitor cells belonged to a Sca-1^+^/CD31^-^ subpopulation, which has been reported to show functional cardiomyogenic differentiation [21]. Sca-1^+^/CD31^-^ CPCs may be promising starting material to produce cells for cardiac regeneration. However, little is known about these cells’ stemness characteristics or differentiation potential after long-term culture in vitro. These features will determine whether it is possible to obtain sufficient seed cells from these rare primary cells. Here, we monitor these features in cultured CPCs, propagating Sca-1^+^/CD31^-^ CPCs over 53 passages. If calculated from one primary isolated cell, with a splitting ratio of 1:3, there would be approximately 2 × 10^14^ cells at P28 and approximately 2 × 10^25^ cells at P53. These quantities would be sufficient to meet the requirements of cardiac tissue engineering.

Stemness characteristics include specific marker expression, proliferation ability, and differentiation potential. In gene expression profile analysis, Sca-1^+^ cardiac cells were positive for the cardiac mesoderm precursor markers Isl-1 and TBX5, and cardiac-specific transcription factors Nkx2.5 and GATA4, but negative for the cardiomyocyte structure gene α-MHC (Figure 4A). Isl-1 is a marker of cardiac precursors in the developing as well as postnatal heart [15], and also is a marker of cells in the secondary heart field. Isl-1^+^ cells are considered to be cardiovascular precursors with the ability to differentiate into the three main cardiac cell lineages [28]. In contrast, Tbx5 is expressed in the primary heart field [29]. Proliferation ability is another important stemness characteristic. Here, Sca-1^+^ CPCs in early, middle and late passages proliferated rapidly during days 1–3 after seeding, with continuous growth that peaked at day 7. These results are consistent with previous reports [17]. There were significant differences in proliferation ability between P7, P28 and P53 during the period of rapid proliferation (Figure 4B). P53 showed the greatest proliferation ability of the three. Tateishi and colleagues have reported that knockdown of Sca-1 transcripts in CPCs, by inactivating Akt, leads to retarded ex vivo expansion and apoptosis. That is, Sca-1 signaling might be essential to promote CPC proliferation and survival [30]. In this study, the purity of Sca-1^+^ cells was highest at P53, which may have contributed to their greater proliferation ability. To test whether cells became tumorigenic over long-term culture, Sca-1^+^ cells were injected subcutaneously into nude mice: in over more than 3 months of observation, no neoplasms appeared.

We tested the differentiation potential of Sca-1^+^/CD31^-^ cells in early, middle, and late stages of culture. After differentiation, Sca-1^+^ cells presented specific marker expression patterns of multiple cardiac cell lineages, such as sMHC, SMA and calponin for smooth muscle, CD31, vWF and VE-cadherin for endothelial cells, and cTNT, cMHC and MLC for cardiomyocytes (Figures 5, 6 and 7). There were no significant differences between early, middle and late passages in differentiation potential. This study nevertheless has some limitations. Although the cells did present cardiomyogenic differentiation potential, the frequency of cardiomyogenic differentiation was lower than expected. Though 5-azacytidine is a classic agent to induce bone marrow-derived cells to efficiently differentiate into cardiomyocytes, it appears less effective in the Sca-1^+^ CPC population [17]. Previous research has shown that cardiomyogenic differentiation of Sca-1^+^ CPCs induced by dexamethasone, dimethylsulfoxide, 5-azacytidine, oxytocine, TGF-β, Wnt1, Wnt5a, BMP2, and FGF4, individually or in combinations, failed to generate mature, beating cardiomyocytes. Only stimulation with BMP2 combined with FGF-4 has been shown to induce the expression of mature cardiac markers [17]. Therefore, we also utilized BMP2 combined with FGF-4 to induce Sca-1+ CPCs for cardiomyogenic differentiation in our research. Regardless of a failure to generate beating cardiomyocytes, the ratio of cardiomyogenic marker expression was still low (Additional file 2: Figure S1A). Further studies are required to identify efficient inducing factors. The function of differentiated cells from those Sca-1^+^/CD31^-^ progenitor cells should also be investigated in the future.

## Conclusions

We have isolated postnatal mouse heart-derived Sca-1^+^/CD31^-^ CPCs by MACS, which can be propagated in vitro over the long-term while retaining stemness characteristics and multiple differentiation potentials. This research may present a possibility that in future clinical studies on cardiac regeneration we can provide a sufficient source of seed cells from little human heart tissue.

## Methods

### Experimental animals and ethics statement

Wild-type ICR mice (3–4 weeks old) and nude mice (3–4 weeks old) were used for cell isolation and subcutaneous cell injection, respectively. All mice were purchased from the Shanghai Laboratory Animal Center (Shanghai Institutes for Biological Sciences, Chinese Academy of Sciences). The Animal Care Committee of Shanghai Children’s Medical Center approved all procedures and protocols. The investigation conformed to the Guide for the Care and Use of Laboratory Animals of the Shanghai Children's Medical Center.

### Cell isolation

Whole hearts were extracted from either male or female ICR mice, split, and washed several times with ice-cold PBS to remove the residual blood. The hearts were minced and digested with 0.1% collagenase type A (Roche) at 37°C for 60 minutes, then filtered through a 40 μm cell strainer (BD Falcon). Half of the filtered cardiac cell suspension was used for flow cytometry (FCM) analysis; the other half was centrifuged at 300 g for 5 min at 4°C to remove the blood cells and debris in the supernatant, then resuspended in cold M-buffer saline (containing 1% BSA; Miltenyi Biotec). After incubation for 5 min on ice (to remove settled cardiomyocytes), the supernatant was collected in a new tube and re-centrifuged. The sample was resuspended and an aliquot taken to determine the cell number. The sample was then centrifuged at 300 g for 10 min at 4°C. The supernatant was aspirated completely, and the cell pellet was resuspended in 90 μl cold M-buffer, followed by incubation with 10 μl FITC-conjugated monoclonal anti-mouse Sca-1 antibody (Miltenyi Biotec) at 4°C for 10 min. The cells were washed 3 times with cold buffer, re-centrifuged, resuspended in 80 μl buffer, then incubated with 20 μl microbeads-conjugated monoclonal anti-FITC antibody(Miltenyi Biotec) for 15 min at 4°C. Cells were then passed through a magnetic cell sorting (MACS) column in a Miltenyi magnetic field to isolate a Sca-1^+^-enriched cell suspension. All manipulations were conducted with aseptic technique and MACS was performed in the dark. Samples of the Sca-1^+^-enriched suspension and the flow-through suspension were subsequently used for flow cytometry. The residual Sca-1^+^-enriched cell suspension was used for further culture. The cell isolation experiment was repeated three independent times, and each time 3–5 mice were used.

### Cell culture

The Sca-1^+^-enriched cells were seeded into 0.1% gelatin-coated 12-well culture plates and cultured in growth medium [16] composed of EBM-2 medium (EGM-2 Single Quots; Lonza) and M199 medium (Gibco) at the ratio of 1:3, plus 10% fetal bovine serum (FBS; Gibco), 1% penicillin-streptomycin (P/S; Gibco), 1% MEM nonessential amino acids (Gibco), and 10 ng/ml basic fibroblast growth factor (bFGF; Roche). Cells were cultured at 37°C in humid air with 5% CO_2_. Cells were given at least 3 days to attach before refreshing the medium and were passaged at 80-90% confluence. Cells were washed with PBS, detached with 0.25% trypsin-EDTA (Gibco), then split at ratios from 1:2 to 1:5. When the cells covered a surface larger than 10 cm^2^, they were converted to cultures in normal medium that contained low glucose Dulbecco’s modified Eagle’s medium (DMEM; Gibco), 10% FBS, and 1% P/S, without bFGF.

### Flow cytometry (FCM) analysis

FCM analysis was performed on the whole cardiac cell suspension filtered through a 40-μm strainer (pre-MACS), the Sca-1^+^-enriched cell suspension (post-MACS) and the flow-through cell suspension (post-MACS) to evaluate the effectiveness of MACS and the purity of the Sca-1^+^-enriched population. The pre-MACS suspension was further divided into two equal parts, one of which was used for a blank control. Other cell preparations were resuspended in 100 or 200 μl buffer and incubated with FITC-conjugated anti-Sca-1 antibody (Miltenyi Biotec) at 4°C for 30 minutes.

To determine whether the expression of surface markers in Sca-1^+^-enriched cells changed during long-term culture in vitro, cells from three passages (P7, P28 and P53) were detached using 0.25% trypsin/EDTA solution. Cells from each passage were counted and divided into several samples (1-5 × 10^5^ cells/sample). For immunofluorescence labeling, each cell sample was resuspended in 100 μl buffer and incubated for 30 minutes at 4°C with PE-labeled anti-Sca-1 (eBioscience), anti-c-kit (CD117; eBioscience), anti-Flk-1 (eBioscience), anti-CD31 (eBioscience), anti-CD45 (eBioscience) antibodies, or APC-labeled anti-CD34 (eBioscience) antibody with dilutions as suggested by the manufacturer. The cells were subsequently washed 3 times with buffer, resuspended in 100 μl cold buffer, then analyzed in a FACSCanto IIflow cytometer (BD Biosciences). Non-stained cells from each passage were used as controls. And this experiment was performed three independent replicates.

### Differentiation assays

Three cell populations (P7, P28, and P53) were cultured in specific differentiation medium for induction into different cardiac cell lineages. Cells were initially seeded at a density of 1 × 10^4^ cells/cm^2^ in normal medium, then converted into specific differentiation medium when they approached 80-90% confluence. For smooth muscle cell induction, cells were cultured in normal medium supplemented with 10 ng/ml transforming growth factor-β1 (TGF-β1; Peprotech) for 10 days. For endothelial cell induction, cells were cultured in EBM-2 medium (Lonza) plus the EGM-2 Single Quots kit (Lonza) but without hydrocortisone, plus 20 ng/ml vascular endothelial growth factor-165 (VEGF_165_, Peprotech), and were grown on fibronectin-coated dishes for 14 days. For cardiomyocyte induction, cells were cultured with DMEM/F12 (Gibco) supplemented with 2% FBS, 2% B27, 100 ng/ml bone morphogenetic protein-2 (BMP-2; Peprotech), and 100 ng/ml fibroblast growth factor-4 (FGF-4; Peprotech) on gelatin-coated dishes for 14 days. In control group, cells were cultured in the same medium for differentiation without growth factors. All the media were changed every 3 days.

### Cell proliferation analysis

Cells (P7, P28, P53) were seeded at a density of 1 × 10^4^ cells/cm^2^ on 24-well culture plates and cultured in normal medium at 37°C in humid air with 5% CO_2_. Cell counting kit-8 (CCK-8; Dojindo) antigen was added to the cell supernatant at the ratio of 1:10 for a 2 h incubation on days 1 (24 hours after initial seeding), 3, 5, 7, and 9. Subsequently, 100 μl of supernatant from each well was carefully transferred to a 96-well plate to measure the optical density(OD) value at a wavelength of 450 nm. It was measured at the five time points with 6 technical replicates of each passage. The living cell number can be indirectly presented by the OD value as they are positively correlated. And this experiment was also performed three independent replicates.

### Analysis of mRNA expression by reverse transcription polymerase chain reaction (RT-PCR)

Total RNA was extracted from P7, P28, P53, differentiated cells, and whole mouse heart tissue using TriZol reagent (Invitrogen), according to the manufacturer’s instructions. Integrity of the RNA samples was verified with a NanoDrop 2000 (Thermo Scientific). Total purified RNA samples were treated with RNase-free DNase (Qiagen) to eliminate DNA contamination and were stored at -80°C until use. RT-PCR was carried out in two steps, and reverse transcription was performed using PrimeScript RTase (TaKaRa). An equal amount of each sample of cDNA was amplified with 2× Taq Master Mix (OFFO BioPharm, Shanghai). The primers used for polymerase chain reaction (PCR) are shown in Additional file 3: Table S2. The reaction was performed with 38 cycles, with denaturation at 95°C for 30s, annealing from 54-70°C for 30s, and extension at 72°C for 30s. After the last cycle, PCR products were run on a 1-2% agarose TAE gel (OFFO BioPharm, Shanghai). Expression levels were evaluated by the intensity of the band stained with ethidium bromide. Quantified values were normalized against the housekeeping gene, GAPDH. The RT-PCR tests were replicated three times.

### Immunofluorescence (IF) staining

Cells from P7, P28, and P53 were analyzed with IF staining for the surface markers Sca-1 and CD31. Induced and control group cells were labeled with IF for cardiac cell lineage-specific markers. Cells were washed with PBS and fixed with 4% paraformaldehyde for 15 min at room temperature (RT), permeabilized if noted with 0.3% Triton X-100 (Sigma) for 20 min at RT, washed with PBS, blocked with 10% donkey or rabbit serum (Multisciences Biotech, China) in PBS for 30 min at 37°C, and then incubated for 2 hours at RT with primary antibodies against Sca-1 (Millipore), CD31 (eBioscience), cardiac troponin T (cTNT; Santa Cruz), cardiac-myosin heavy chain (cMHC; Abcam), smooth muscle actin (SMA; Epitomics), smooth muscle-myosin heavy chain (sMHC; Abcam), or calponin-1 (Abcam). After rinsing with PBS, cells were subsequently incubated with Alexa Fluor 488-conjugated donkey or rabbit-originated secondary antibodies (Life Technologies, Invitrogen) or PE conjugated streptavidin (eBioscience) for 30 min at RT. Nuclei were counter-stained with DAPI (1:1000; Enzo) in PBS for 1 min at RT. Immunostaining was observed and photoed by an inverted fluorescence microscope (Leica DMI3000B, Germany) and analyzed by Image-Pro Plus 7.0 software (Media Cybernetics, America).

### Tumorigenic assays

2 × 10^6^ cells from P7, P28, and P53 were injected subcutaneously into one dorsal side of severe combined immune deficiency (SCID) mice. As a positive control, an equal quantity of mouse R_1_ embryonic stem (ES) cells were inoculated subcutaneously into the opposite dorsal side of the same mice. Details of the cell type, cell concentration, injection volume and injection points are shown in Additional file 1: Table S1.

### Statistical analysis

All data are expressed as mean ± SD. Significance between two comparisons was evaluated by independent-samples *T* test. Significance between multiple comparisons was evaluated by one-way ANOVA. Bonferroni post-hoc tests were used to identify differences. Statistical values were calculated using the SPSS 17.0 software. A value of P < 0.05 was considered statistically significant.

## Competing interest

The authors declare that they have no competing interest.

## Authors’ contributions

Conceived and designed the experiments: HW HC WF ZX. Performed the experiments: HW HC BF XW XH RH MY. Analyzed the data: HW HC WW WF. Drafted the manuscript: HW HC WF ZX. All authors read and approved the final manuscript.

## Supplementary Material

Additional file 1: Table S1 Tumorigenic Assay. Click here for file

Additional file 2: Figure S1 Quantitative analysis of differentiation potential of subcultured cells from Sca-1^+^-enriched populations into cardiac cell lineages in vitro. A, cMHC or cTNT positive cells were calculated after induction to cardiomyocyte-like cells. (n = 10). B, SMA, sMHC or calponin positive cells were calculated after induction to smooth muscle-like cells. (n = 10). C, CD31 positive cells were calculated after induction to endothelial-like cells. (n = 10). The positive rate was presented as ratio of positive cell number to total cell number (*p < 0.01 vs control). Click here for file

Additional file 3: Table S2 Primers used for reverse transcription PCR. Click here for file
