# Supplementary material for: Isolation and characterization of a Sca-1+/CD31- progenitor cell lineage derived from mouse heart tissue
Source: BMC Biotechnol. 2014 Aug 9;14:75. doi: 10.1186/1472-6750-14-75 (PMC4133720; doi:10.1186/1472-6750-14-75)
Supplement: Additional file 1: Table S1 — Tumorigenic Assay. [file 1472-6750-14-75-S1.docx]

**Table S1:** Tumorigenic Assay

| **cell type** | **cell concentration** | **Injection volume per point** | **injection points** | **newoplasm number** | **tumorigenic rate** |
| --- | --- | --- | --- | --- | --- |
| CPC (P7) | 2×1000000/100μl | 100μl | 6 | 0 | 0% |
| CPC (P28) | 2×1000000/100μl | 100μl | 6 | 0 | 0% |
| CPC (P53) | 2×1000000/100μl | 100μl | 6 | 0 | 0% |
| ES (R1) | 2×1000000/100μl | 100μl | 18 | 18 | 100% |
