# Supplementary figures and images for: Isolation and characterization of a Sca-1+/CD31- progenitor cell lineage derived from mouse heart tissue
Source: BMC Biotechnol. 2014 Aug 9;14:75. doi: 10.1186/1472-6750-14-75 (PMC4133720; doi:10.1186/1472-6750-14-75)

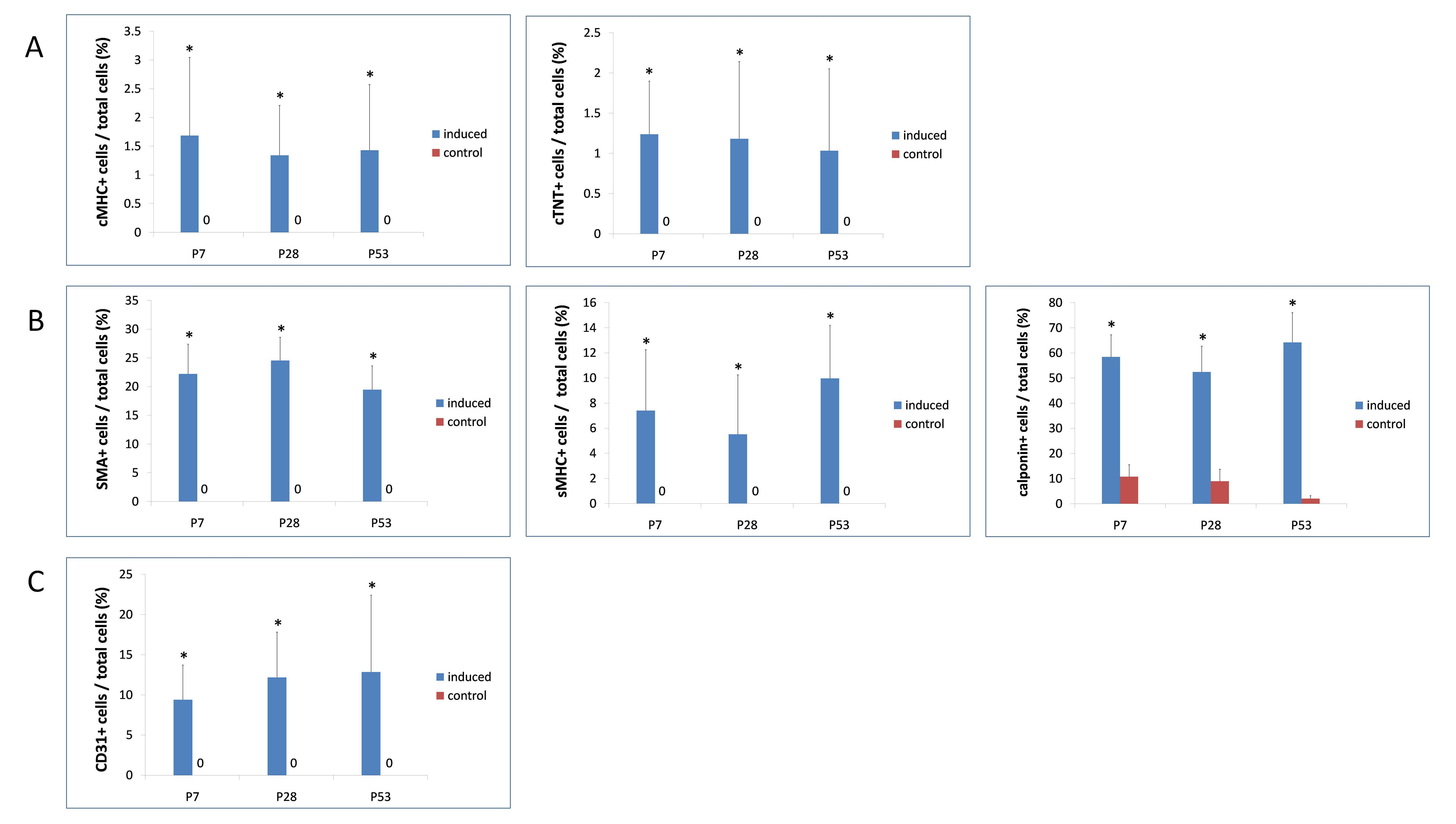

Supplement: Additional file 2: Figure S1 — Quantitative analysis of differentiation potential of subcultured cells from Sca-1+-enriched populations into cardiac cell lineages in vitro. A, cMHC or cTNT positive cells were calculated after induction to cardiomyocyte-like cells. (n = 10). B, SMA, sMHC or calponin positive cells were calculated after induction to smooth muscle-like cells. (n = 10). C, CD31 positive cells were calculated after induction to endothelial-like cells. (n = 10). The positive rate was presented as ratio of positive cell number to total cell number (*p < 0.01 vs control). [file 1472-6750-14-75-S2.tiff]
