# Supplementary material for: Isolation and characterization of a Sca-1+/CD31- progenitor cell lineage derived from mouse heart tissue
Source: BMC Biotechnol. 2014 Aug 9;14:75. doi: 10.1186/1472-6750-14-75 (PMC4133720; doi:10.1186/1472-6750-14-75)
Supplement: Additional file 3: Table S2 — Primers used for reverse transcription PCR. [file 1472-6750-14-75-S3.docx]

**Table S2:** Primers used for reverse transcription PCR

| **Genes** | **Sense Primer** | **Anti-sense Primer** | **Product Tmpt** | **Product Length** |
| --- | --- | --- | --- | --- |
|  |  |  | **(℃）** | **（bp)** |
| GAPDH | GACATCAAGAAGGTGGTGAAGC | TCCACCACCCTGTTGCTGTA | 60 | 208 |
| Nanog | GTTCACGCCATTCTCCTG | ACGAATACATCTTCATCACCAAT | 61.7 | 349 |
| TERT | CTCAGACTTCAAGACCAT | GGATGGACTATTCCTATGT | 65.2 | 354 |
| ISL-1 | GTCATTGCCTTGCCAAACAG | AATCCCGAGACGCTTGTG | 60.5 | 331 |
| TBX5 | CCAACCTTCCAAACCTCCATCA | CACACTCAGCCTCACATCTTACC | 59.4 | 472 |
| GATA4 | CCTTCGTTCACCGTGTCA | TGTCTTAGCAGTCGTCTTCTT | 63.9 | 352 |
| NKx2.5 | GCCGCCAACAACAACTTC | GCATAATCGCCGCCACAA | 65.6 | 310 |
| MEF_2_C | GAGCAATCCAAGCCACATATCT | ACCATCTGAAGCAATCCAAGTC | 59.4 | 238 |
| α-MHC | ATGCGGGTCACGGCGCCCCGAAC | TGGCCTTGCAGATCTGTGTCTCCCG | 56 | 298 |
| β-MHC | CTGGCACCGTGGACTACAAC | CGCACAAAGTGAGGATAGGGT | 56 | 268 |
| MLC-2a | AGGCACCATTCAGGAAGATTACC | AGAAGAGGCAAAGCTGTGAAACTA | 61.3 | 294 |
| MLC-2v | GTGCTGAAGGCTGATTAC | AAGAAGATGGAGGTGGATAA | 63.5 | 303 |
| SMA | ATGATGCTGATGCTTTGGGAAGTA | CAACTGCCTCACCACCGT | 60.5 | 399 |
| Calpeuin | GGCAAGGACAGTGGAGAG | GGCTGAGGCTGGAGAATC | 64.6 | 362 |
| FLK-1 | CGAGTTGGGCTAAAGTAGAGTTC | TCAGACATATCACATCAGGACAGA | 62.1 | 274 |
| CD-31 | TCAACTTCAAGCTCCTAA | CCACTCAGACTTTATTCAAA | 63.7 | 245 |
| vWF | TAAGTCTGAAGTAGAGGTGGATA | GGCACAAGAGCAGAACAT | 64 | 322 |
| VE-Cadherin | TTCTCTGTCTACTCCTTATCC | GTTGACTGATGCCACTTC | 61.6 | 270 |
